# Standardization of *In Vitro* Studies for Plasmonic Photothermal therapy

**DOI:** 10.1021/acsnanoscienceau.3c00011

**Published:** 2023-07-17

**Authors:** Helena Villuendas, Clara Vilches, Romain Quidant

**Affiliations:** † Nanophotonic Systems Laboratory, Department of Mechanical and Process Engineering, ETH Zürich, 8092 Zürich, Switzerland; ‡ ICFO − Institut de Ciències Fotòniques, the Barcelona Institute of Science and Technology, 08860 Castelldefels, Barcelona, Spain

**Keywords:** Hyperthermia, Plasmonic photothermal therapy, Reproducibility, In vitro, Reporting standards

## Abstract

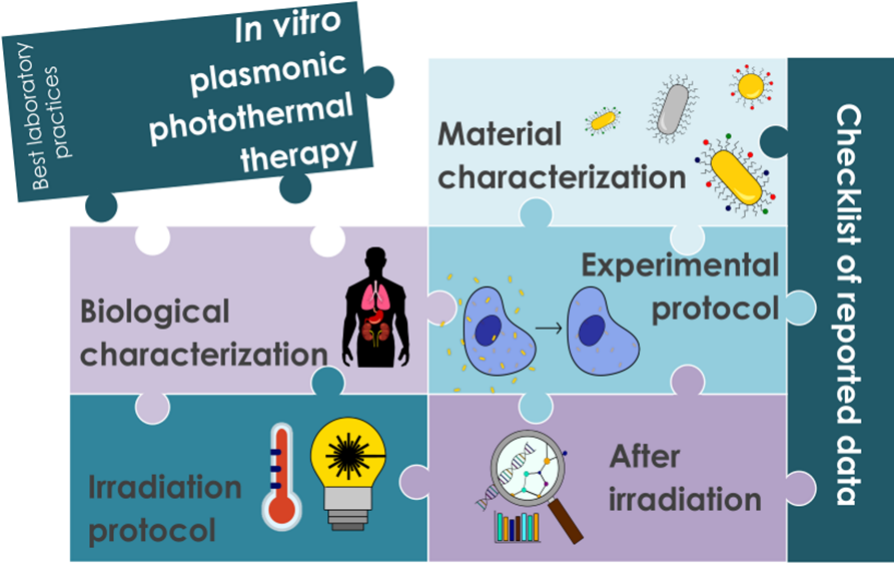

Lack of standardization is a systematic problem that impacts nanomedicine by challenging data comparison from different studies. Translation from preclinical to clinical stages indeed requires reproducible data that can be easily accessed and compared. In this work, we propose a series of experimental standards for *in vitro* plasmonic photothermal therapy (PPTT). This best practice guide covers the five main aspects of PPTT studies *in vitro:* nanomaterials, biological samples, pre-, during, and postirradiation characterization. We are confident that such standardization of experimental protocols and reported data will benefit the development of PPTT as a transversal therapy.

Clinical translation in nanomedicine faces considerable barriers that impact the application of nanomaterials for human use. Drug regulatory agencies provide licensing for diagnostic and therapeutic nanomaterials, setting high standards to guarantee both their safety and effectiveness. One of the systematic problems that influence nanomedicine is the lack of standardization, which challenges contrasting data from different studies.^1−3^

Standardization diminishes the commercial, academic, and societal concerns^2^ by creating experimental protocols that produce robust and reproducible data that are easily accessible by researchers and allow comparisons between different studies.^1,4^ Obtaining and presenting data in a comprehensive manner improves the validity of results, accelerating approval from regulatory agencies. Guidelines that outline the minimum information to be reported improve our understanding of acquired data. The MIRIBEL guidelines for bionano experimental literature^2^ is an excellent example of how methodical approaches for material characterization, risk assessment and experimental design improve the development of novel technologies for biomedical use.

In the specific case of plasmonic photothermal therapy (PPTT), lack of standardization is one of the main hindrances for the development of PPTT-based therapies, as pointed out by Sharifi et al.^5^ Albeit not being the only obstacle of PPTT to reach clinical practice, following a methodical approach can only encourage researchers to create robust and comprehensive data. Standardization will facilitate the creation of systematic studies to evaluate different types of nanoparticles, biological systems–their interaction–as well as to identify sources of variability and increase reproducibility on *in vitro* research by keeping reference standards.^6^ Systematic studies of *in vitro* PPTT will push the field forward by addressing concerning questions about biocompatibility, delivery, and effectivity.

PPTT has emerged as a complementary technique for cancer treatment by locally combining plasmonic nanoparticles and near-infrared laser light to locally increase the temperature and impair the cellular viability. The potential of this therapy has been repeatedly proven *in vitro* and has been performed with success in clinical human pilot studies.^7^ However, it still presents difficulties to be further validated *in vivo* due to the limitations animal experimentation imposes, such as increased costs and ethical concerns. *In vitro* studies have a limited ability to replicate the complexity of *in vivo* environments; ergo, results may not accurately reflect what occurs in organisms. Moreover, since researchers use different protocols, cell lines, and nanoparticles, data and conclusion are difficult to compare.^8^

In this work, we propose a series of best laboratory practices along with a checklist about minimum data to be reported (Figure 1 and Table 1). We present five different aspects of PPTT that can be easily improved by applying these standards in combination with the fundamental criteria presented by Faria et al.^2^ Standardization of reporting data and minimal requirements regarding experimental protocols can be a milestone for the development of PPTT.

**Figure 1:**
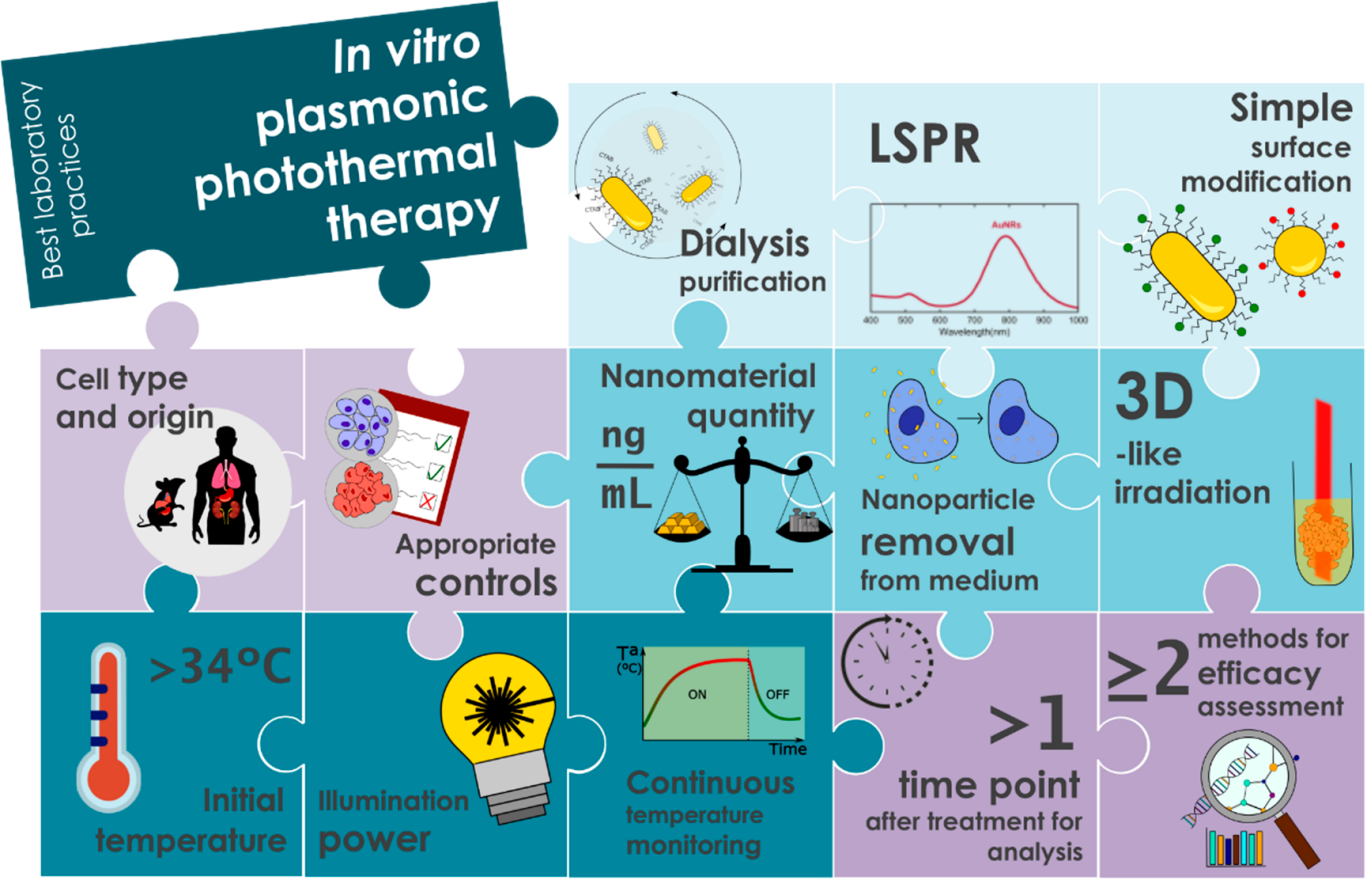
Schematic representation of the main key aspects to ensure meaningful and reproducible data for *in vitro* PPTT.

**Table 1:** Summary and Checklist of Parameters to Be Reported on *In Vitro* PPTT Experiments^a^

parameter	representative units	information
**material characterization**		
method of purification		dialysis, ultracentrifugation...
conjugation-stabilizer		
laser wavelength	nm	
nanoparticle LSPR	nm	
nanoparticle type and ligand information		MIRIBEL guidelines^b^
**biological characterization**		
cell culture details		MIRIBEL guidelines^b^
cell type and origin		epithelial, human, cancerous...
appropriate controls		
**experimental protocol**		
nanomaterial quantity	ng/mL, μg/μL	amount of material in the nanoparticle samples
time of incubation	min, h	
nanoparticle removal prior to irradiation	yes, no	
irradiation dimension		2D, 3D, organoid, suspension...
type of well plate		flat bottom, U-bottom, cuvette...
time between administration and therapy	min, h	
**irradiation protocol**		
temperature		
initial temperature	°C	
final temperature	°C	
temperature increase	°C	difference between initial and final temperature
temperature recording		
type		IR thermal camera, probe...
time period		continuous, intermittent...
position		position regarding the sample (top, lateral, bottom...)
baseline and cool-off	s, min	temperature recorded before and after irradiation
laser		
type		collimated, divergent
position	cm	position and distance to the sample (top, lateral, bottom...)
beam diameter/area	mm, mm^2^	
power density	W/cm^2^	
time of irradiation	s, min	
**after irradiation**		
method of assessment		techniques used to assess viability, toxicity...
time after treatment	min, h	time between the end of irradiation and viability assessment

a This table provides a summary of the parameters to be included on *in vitro* PPTT experiments. We provide representative units to use when reporting results.

b MIRIBEL guidelines^2^ complete the proposed table for characterization of bionano interactions.

## Material Characterization

### Nanoparticle Purification

The goal of nanoparticle purification is to eliminate the cytotoxic fraction, remove medium-related impurities, and obtain sample homogeneity for enhanced biocompatibility. Seed-mediated growth synthesis is one of the most versatile methods to produce gold nanoparticles (AuNPs). A key element is the use of cetyltrimethylammonium bromide (CTAB), which works as a stabilizing agent during synthesis. CTAB has shown high cytotoxicity levels and several strategies have been adapted to remove it from nanoparticles after synthesis.^8^

Ligand exchange and functionalization increases biocompatibility and reduces clusters, but CTAB remains in the medium and washes by centrifugation do not eliminate it completely. To further improve biocompatibility of nanoparticles, dialysis can be used to eliminate CTAB in solution.^9^ The dialysis membrane acts as a net that traps the nanoparticles inside, allowing CTAB to flow outside, reducing the overall toxicity. Moreover, dialysis can be used to eliminate organic solvents used during functionalization and unbound moieties of surface functionalization.

Nanoparticle purification can also be achieved by other high-yield processes such as tangential flow filtration and salt-/buffer-exchange columns, among others. Each method presents distinct advantages and disadvantages to remove medium-related impurities in nanoparticle solutions.^10^ The final choice will depend on the specific requirements of the nanoparticles. Moreover, considerations of time, cost, and scalability are crucial to select the most appropriate technique.

### Simple Surface Modifications

Surface modifications are essential in most types of AuNPs to increase their biocompatibility. Nanoparticle functionalization has three main objectives: targeting, increasing colloidal stability, and reducing cytotoxicity. Thousands of molecules can be conjugated to nanoparticles, from inorganic moieties to antibodies, to produce multifunctional nanoparticles, e.g., to target specific tissues. Complex nanoparticles can have increased costs of production and less consistent large-scale batches, hindering commercialization and approval by regulatory agencies.^5,11−13^ When combining a nanoparticle with a functionalization, targeting or drug, it is necessary to test the toxicity of all excipients in both bound and released states, including all potential combinations.^12^ The more complex the nanoparticle, the more steps regulatory agencies will require. Simple surface modifications overcome this, as well as having more scalable synthesis, less time-consuming, and more affordable formulations.

Wilhelm et al.^14^ performed a meta-analysis of 117 research papers regarding *in vivo* delivery of nanoparticles to target tissues. Findings showed that, on average, there was only 1% delivery efficiency of AuNPs in target cells. A head-to-head comparison between nanoparticles with passive or active targeting revealed a lack of significant differences in tumor accumulation. This is partially caused by the formation of a protein corona when the nanoparticle interacts with a biological fluid, a dynamic process that changes the physicochemical properties of the nanoparticle and their interaction with the cell membrane.^15^ Characterization of nanomaterial behavior in contact with a biological fluid cannot be fully assessed *in vitro*.

### Localized Surface Plasmon Resonance

Plasmonic nanoparticles have the ability to strongly absorb light at their localized surface plasmon resonance (LSPR), defined by the constitutive material and geometry of the particle. For instance, nonsymmetrical nanoparticles like nanorods (AuNRs) feature different absorption bands (longitudinal and transversal). Modifying the aspect ratio of AuNRs is used to tune the longitudinal LSPR to the near-infrared region (NIR),^16^ to match the biological optical window, ensuring minimum light absorption by the tissue.^17^ Therefore, measuring the detuning of the laser wavelength with respect to the LSPR peak of the nanoparticles is important, as it directly affects the efficiency of light-to-heat conversion.

## Biological Characterization

### Cell Type and Origin

Cell line selection for *in vitro* experimentation must account for their different behaviors. Immortalized cell lines will conduct themselves differently to primary cell lines and have unique limits to what can be characterized with them. The choice of cell type (epithelial, fibrotic, tumoral...), source of origin (lung, kidney, skin...), and organism (human, mice...) affects the outcomes of *in vitro* PPTT and determines follow-up research based on these results. Cell type and its manipulation have an important effect on the outcomes of *in vitro* variability,^1,6,18^ which will impact interpretation of the PPTT data. As an example, nanoparticle internalization depends not only on the properties of the nanomaterial and experimental protocol, but also on the cell type, observing trend differences between type and origin of cells.^19,20^

All in all, we consider more precaution should be taken when selecting the cell line to study *in vitro* PPTT. Data comparisons and future research will depend on the cell lines chosen, and therefore, a justification of why one is selected would be essential to fully comprehend the outcomes of the therapy.

### Appropriate Controls

The use of appropriate controls ensures the validity and robustness of the experiment outcome.^6^ Studies across different cell types and correlation with *in vivo* data provides the consistency needed to translate to clinical settings.^21^ PPTT aims to specifically eliminate upon illumination malignant cells that have internalized nanoparticles. However, nonmalignant cells will also be exposed to both laser light and nanoparticles. It is important to study the influence of both factors and their combination will have on nonmalignant cells. Noncarcinogenic cell lines used as control should be from the same type, tissue, and organism to confirm the data robustness and safety of the nanomaterial.

Moreover, during systemic *in vivo* administration of nanoparticles, only ≈1% of the injected dose reaches target cells,^14^ meaning there is a lot of off-target interactions of nanoparticles with cells.^12^ It is important to conduct experiments not only in malignant and nonmalignant cell lines but also on cells from different tissues.^3,18^ This will provide information regarding the effectivity of PPTT in different cancers, identify possible side effects on off-target cells, and set limits for light and nanoparticle dose safety.

## Experimental Protocol

### Nanomaterial Quantity

Cytotoxicity of nanomaterials in PPTT is studied usually by performing sequential dilutions of nanoparticle concentration and studying the viability after exposure, by one or several methods, at different end points. Concentrations are usually reported in molar concentration or as optical density, which becomes an obstacle to compare different studies, as size, shape, material composition, or other factors could also be responsible for the cytotoxicity of nanoparticles.

Lack of standardization regarding nanoparticle concentrations makes it difficult to obtain consensus regarding cytotoxicity of plasmonic nanoparticles, as already reported by Jauffred et al.^22^ Providing the concentration of the nanomaterial (e.g., nanograms per milliliter of gold in AuNRs) is essential to assess the biocompatibility of nanoparticles.

AuNRs are one of the most used nanoparticles in PPTT owing to their high biocompatibility, stability, and photothermal efficiency. For AuNRs, cytotoxicity increased with the total gold concentration in the suspension, and was found to be independent of the shape.^23^ Defining the nanomaterial quantity in a nanogram per milliliter format helps contrast data between researchers and determine which factors (size, shape, material, etc.) govern the toxicological events of plasmonic nanoparticles. Similarly, internalized doses of the nanomaterial should be specified to obtain direct definitions of toxicity of nanoparticles, in contrast to exposure doses.^1^

### Nanoparticle Removal from the Medium

In most *in vivo* PPTT experiments, it is important that cells can incorporate the nanoparticles and that they are equally distributed inside tumors. Moreover, it is critical that internalized nanoparticles can be irradiated externally. Efficacy of PPTT depends on the combination of biocompatibility, cellular uptake, and heat generation efficiency. When studying *in vitro* PPTT, cells are exposed to plasmonic nanoparticles and later irradiated. Between these two steps, most protocols require several washes to remove nanoparticles that are not incorporated by cells. Skipping their removal from the medium can have an impact on temperatures achieved, consequently leading to incorrect conclusions about the efficacy of PPTT to eliminate targeted cells.

Some protocols omit the washing step and irradiate cells in a suspension of nanoparticles. This leads to temperature measurements of nanoparticle suspensions and not of internalized nanoparticles. Specifying the removal of nanoparticles prior to irradiation is key for ensuring reproducibility between the experiments and detecting differences in the final temperatures achieved.

### 3D-like Irradiation

One of the main challenges of *in vitro* research is the lack of dimensionality. Nowadays, this challenge can be overcome by working with spheroids, organoids or cell scaffolds. However, these techniques can be difficult to master, and manipulation of cells is more complicated.

In PPTT *in vitro*, most research is performed under 2D settings, where a single layer of attached cells is being illuminated. An alternative to 2D irradiation was described by Yang et al.,^24^ in which cells are grown and incubated with nanoparticles in standard 2D conditions, but prior to irradiation, they are detached from the surface and illuminated in suspension. Irradiation of a 3D cell arrangement increases collective thermal effects, hence increasing the homogeneity of the temperature profile as well as maximum temperature increments.^25^ Moreover, heat dissipation from the surrounding environment is reduced. Experiments on droplets of medium containing a high concentration of cells better mimic an *in vivo* situation where cells are in a disorganized distribution and cells are not uniformly irradiated. The effect of light and temperature is better reproduced than for 2D attached cells, where a thermal gradient to the outskirts of the irradiated area can impair the viability study after treatment. It is also important to consider that the use of suitable cell lines for 3D culture is important, as cells can have different resistance to external sources of stress, which may affect viability.^26^

Finally, 3D cell arrangements increase the number of cells irradiated and their density, which in turn influences the accumulation of signaling molecules (such as cytokines, growth factors and proteins) that influence cellular biology.^6^ Besides, spatial organization and cell–cell interactions differ from 2D models, consequently impacting the outcomes of the therapy. The creation of 3D models and phantoms that mimic tissue environments are really useful to study laser penetration and obtaining more *in vivo* relevant temperature measurements. Slowly moving toward 3D cell culture methods increases the predictability of *in vivo–in vitro* comparisons.^27^

## Irradiation Protocol

### Initial Temperature

Cell culturing requires, in most mammalian cell lines, maintaining a temperature of 37 °C for optimal cell growth. Nonetheless, typical laboratory temperatures are usually between 20 and 25 °C and cells are commonly manipulated for prolonged periods of time in mild hypothermia conditions, hence influencing the cellular stress response.^28^

PPTT relies on increases in temperature to decrease cellular viability by introducing cells in a hyperthermic environment in a controlled manner. It is essential, therefore, that irradiated cells are maintained in a homeothermic state closer to 37 °C; therefore, increases of temperature achieved during light-to-heat conversion are biologically and therapeutically meaningful by reaching temperatures over 42 °C. Cells that are originally at room temperature eventually experience lower temperatures when irradiated. Any impact on viability could be the only effect of light irradiation and not the temperature increase. By maintaining a starting temperature over 34 °C, all starting conditions are not in mild hypothermia; hence, the stress response only starts upon irradiation.

### Illumination Power

When it comes to illumination, relevant parameters are laser power, dosage, and irradiation time.^12^ Laser power and laser beam diameter indicate the amount of energy per unit area (W/cm^2^) delivered to the cells. Providing power intensity without informing on the illuminated area or beam diameter can lead to confusions and misleading data.

The distance of the light source from the sample can affect the efficacy of the treatment. Indeed, in the case where a noncollimated laser beam is used to illuminate the sample, it becomes highly important to determine the real light power experienced by the cells. Control of the laser beam power enables researchers to precisely control temperature generation in cell lines,^29^ to study similarities and differences of heat generation efficacy and the outcomes it has on cells.

### Continuous Temperature Recording

Light-induced heat generation in plasmonic nanoparticles can be measured by using thermal imaging or temperature probes. Temperature monitoring allows us to study the photothermal stability of the nanomaterial and the photothermal conversion efficiency. Bulk measurements of temperature of nanoparticles in a concentrated solution are not equivalent to measuring the temperature increase arising from internalized nanoparticles in cells. Specifying the removal of nanoparticles before irradiation is important in these cases, as different uptake processes by different cell lines exposed to the same nanoparticle concentrations will lead to different degrees of internalization and hence different hyperthermia levels. Temperature estimation in nanoparticle suspensions leads to false assumptions and has an impact on the proper interpretation of results.

Additionally, continuous real-time recording of temperature provides a better understanding of heat generation and insight into differences between irradiated cells. Indeed, continuous measurements help identify laser power settings that do not increase temperature in absence of nanoparticles and detect the appearance of temperature gradients^30^ of cells in a 2D cell arrangement. The collection of these data, together with viability assessments after treatment, increases the possibility to tune temperatures, reaching and maintaining certain values for determined periods of time via feedback mechanisms.

## After Irradiation

### More than One Time Point for Analysis

Broadly speaking, cell death is classified as necrosis and apoptosis. The former is a mechanism that occurs almost immediately after the heat-induced damage; the latter leads to a delayed demise. An increase of temperature initiated by PPTT can cause both mechanisms to be activated depending on the power, exposure time, and heating dose. Cell death is a dynamic process, and evaluating viability at a single time point can miss events occurring on the cellular level. Studying at several time points the molecular mechanisms activated after treatment provides more reliable data and crucial information to differentiate between necrosis, apoptosis, ferroptosis, pyroptosis, and other cell death types. Findings in an experiment performed with radiofrequency ablation, a noninvasive thermal treatment for hepatocellular carcinoma, showed how different temperatures during different exposure times results in different patterns for early and late apoptosis in irradiated cells.^30^ Characterization at more than one time point provides new insights into cytotoxicity of nanomaterials and new possibilities of PPTT.

In this line, analyzing the viability of different cell lines after *in vitro* PPTT, we observed different levels immediately and 24 h after irradiation. The drops in viability after 24 h of irradiation were not equivalent between cell lines, highlighting the importance of evaluating cellular mortality at more than one time point, and in more than one cell line.^20^

### Two or More Methods to Assess Cell Viability

Different cell death mechanisms activate different molecular machinery; therefore, evaluating cellular viability with more than one readout system is as important as assessing viability at different time points.

Complementary assays that provide insight into different cellular parameters can be used to evaluate the activation of one cell death mechanism over another. It is essential to identify the information provided by the viability assay, as different experimental readouts can be interpreted in various ways.^6^ The most common assays measure metabolic active cells (MTT, WST-1, and ROS generation assays) or membrane integrity (Trypan Blue staining and LDH assay). Integrating methods that assess different end points simultaneously will provide a more comprehensive readout and can reveal discrepancies that could otherwise not be observed.^31^ It is critical to systematically use multiple methods to assess viability at multiple time points.

## Conclusions

As research in nanomedicine is foreseen to keep growing, involving new nanoparticles and drugs toward improved treatments, establishing criteria on experimental protocols should simplify contrasting data sets and allow for greater reproducibility of results. The adoption of these best practices can be generalized to other diseases beyond cancer, allowing PPTT to benefit a broader range of clinical scenarios. Likewise, reporting the items enumerated in the checklist will allow a better comparison of results and more synergetic contributions from different laboratories.
